# Consumption

**Published:** 1890-08-30

**Authors:** 


### The Hospital

A Weekly Journal of

Science, Medicine, Nursing, and Philanthropy.

*For Hospitals, Asylums, Medical Practitioners, Students, Nurses, Families, Parishes, Congregations, and the Charitable Public.*

Vol. VIII.—No. 205.] [August 30, 1890.

### Consumption: A Step Forward.

"Truth" informed its readers last week that Koch had discovered a method of destroying the bacillus of phthisis. Many people, therefore, concluded that a cure for consumption had at last been certainly discovered. The conclusion of sanguine readers was not unnatural. But there is a long interval between the discovery of a substance that will destroy the phthisis bacillus within the human organism, and the certain cure of persons languishing in consumption. All those interested in the subject should strive to understand clearly the state of medical knowledge with regard to this particular disease at the present time. It is known that a bacillus is a constant occupant of phthisical lungs: it is believed by some competent observers that the bacillus is one of the causes of phthisis: it may be believed by a very few that it is the chief or even the only cause. But the best medical opinion in this country does not hold that the bacillus is the sole or even always the chief cause of the destructive process in the lung tissue which ends in the victim's death.

In a brief preface to the new edition of the soberest and ablest of all the recent English works on pulmonary consumpton, Dr. Theodore Williams makes two statements, which are of great importance as expressing the mature judgment of both himself and his father, the late Dr. C. J. B. Williams. The first statement bears upon the cause of phthisis. Dr. Williams says: "The tubercle bacillus, the great discovery of Koch, may truly be called the septic element of consumption, though others may exist; and it may be said to exercise a powerfully corrupting influence on the blood and lymph of the body, after the first infective centre has been established." The second statement has reference to treatment. It is this: "My present view of the treatment of consumption is, that while we enter on a life and death struggle with our enemy, the tubercle bacillus, to destroy him in the nest he has made for himself and to eject him from the living patient, we must never omit prophylactic and anti-phthisical measures, which may render his attack harmless, or, if he has effected a lodgment, may confine him to the outwork and prevent his entry into the citadel, and as a proof that this can be done we may point to the remarkable success which has attended such measures, and specially the so-called 'mountain cure.' The outlook of the future of consumption is decidedly hopeful, for there is little doubt that much of the disease is due to preventable causes, which the coming reign of hygiene will sweep away, and that in many cases the disease will be nipped in the bud by a combination of anti-phthisical and bacillicide treatment."

These statements enable any intelligent reader to understand the mental attitude of phthisis specialists in this country, and also furnish the means of forming a rational judgment on Koch's present position. The great German bacteriologist evidently aspires to be a practical physician, as every bacteriologist should. Those who saw and heard him at the International Medical Congress just held were favourably impressed by his personality. He is no mere youth confident in inexperience and aggressive in ambition, but a man in the prime of life and at the height of his intellectual vigour. Koch appears to have satisfied himself that the tubercle bacillus is not merely associated with pulmonary phthisis, but is at least the chief even if not the only cause of it. That conclusion we do not hold to rest upon a sufficiently wide induction. But, agreeing with Koch on this point for the moment, we will consider what are the prospects of successful treatment. If it be granted that the tubercle bacillus is the only cause of phthisis, it must follow that unless the destructive process in the lung has been carried so far as to render the organ incapable of recovering something of its normal structure and of performing its natural function as the oxygenator of the blood, the death of the bacillus should not only suffice to stop the phthisical process, but also to admit of the patient's restoration to health and to a limited degree of vigour. What, then, are the prospects opening before medicine of the destruction of the bacillus within the human body? Non-professional readers should understand that it is one thing to destroy the bacillus in an artificial culture outside the body, but quite another to destroy it in its semi-natural habitat within the lungs. As showing how easy it is to do the former it is only necessary to mention that sunlight alone will kill the tubercle bacillus in an artificial culture in a few hours, and even in a few minutes if the culture be sufficiently thin. Nay, more, ordinary daylight, without sun at all, will kill the organism in six or seven days.

It is proved beyond doubt that daylight, and even bright sunlight, are powerless against the bacilli in the human lung, except perhaps in the very initial stages of their destructive work. Not only does the sun, however, destroy artificial cultures quickly and effectively, but quite a large number of substances have the same effect. Among these are several ethereal oils; some aromatic compounds, such as Naphthylamin, Paratoluidin, and Xylidin; certain of the so-called tar-dyes, such as methyl blue, chinolin yellow, gentian violet, and others; among the metals, the vapour of mercury, and certain compounds of silver and gold. One substance, the cyanide of gold, is especially powerful, and checks the growth of cultivated bacilli even when it is diluted to a two-millionth part of its normal strength. But, strange and perplexing as the experience must have been, every one of these substances proved in the hands of Koch to be absolutely without effect when tried on tuberculous animals.

Happily the experimenter did not allow himself to be discouraged by these manifold failures. Continuing his experiments, he at last hit upon a substance which was effectual in checking the growth of tubercle bacilli, not only in artificial cultures but in the bodies of living animals. This is an extremely significant step in advance. The animals experimented upon were guinea pigs, and the experiments were of two kinds, prophylactic and curative. Certain healthy guinea pigs were placed under the influence of the bacillicide. They were then inoculated with the tubercle virus, but proved to be quite impervious to its attacks. In the curative experiments guinea pigs suffering from general tuberculosis in a high degree were treated with the bacillicide, with the result that the morbid processes were brought to a complete standstill, whilst the body was in no way injuriously affected.

There the matter stands at present. No experiments appear to have been tried on man as yet. Koch does not tell us what the bacillicide is which has produced such wonderful results. He will doubtless begin with man at the earliest possible moment. If his bacillicide should then prove effective; if the tubercle bacillus in the human lung should be destroyed and its ravages stayed; if, as a result, the injured lung should return to its normal condition and the natural health should be restored, the greatest discoverer of the nineteenth century and the noblest benefactor of his kind will stand revealed in the German physician. Who is there in all the wide world who will not wish this doctor and man of science "God speed"?

